# Supplementary material for: Live-Cell Analysis of Human Cytomegalovirus DNA Polymerase Holoenzyme Assembly by Resonance Energy Transfer Methods
Source: Microorganisms. 2021 Apr 26;9(5):928. doi: 10.3390/microorganisms9050928 (PMC8146696; doi:10.3390/microorganisms9050928)
Supplement: Supplementary file 1 [file microorganisms-09-00928-s001.zip › microorganisms-1189137-suplemmentary.pdf]

Live-cell analysis of Human Cytomegalovirus DNA polymerase holoenzyme assembly by resonant energy transfer methods

Veronica Di Antonio[a*], Giorgio Palù[a], Gualtiero Alvisi[a#]

[a] Department of Molecular Medicine, University of Padua, Italy

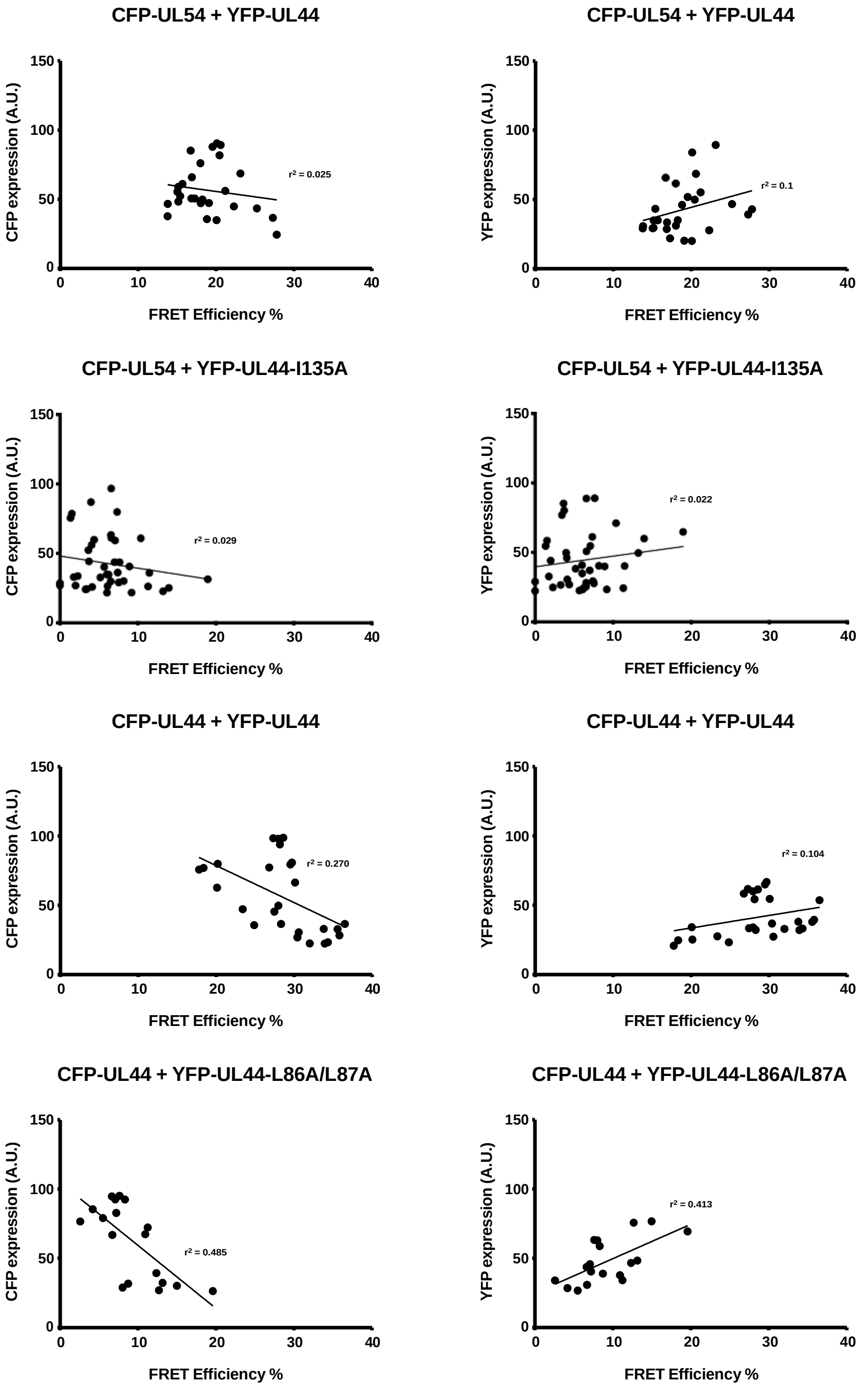

Supplementary Figure S1. Specific residues are involved in UL44 dimerization *in cells*. HEK293T cells were seeded on glass coverslips and transfected with the indicated expression plasmids. 48h p.t., cells were fixed and processed for IF, before being analyzed by FRET acceptor photobleaching to calculate FRET efficiency of individual cells relative to each indicated FRET pair, which is plotted against the respective CFP (*left panels*), and YFP (*right panels*) expression level. The r2 calculated for each lineal regression is shown.

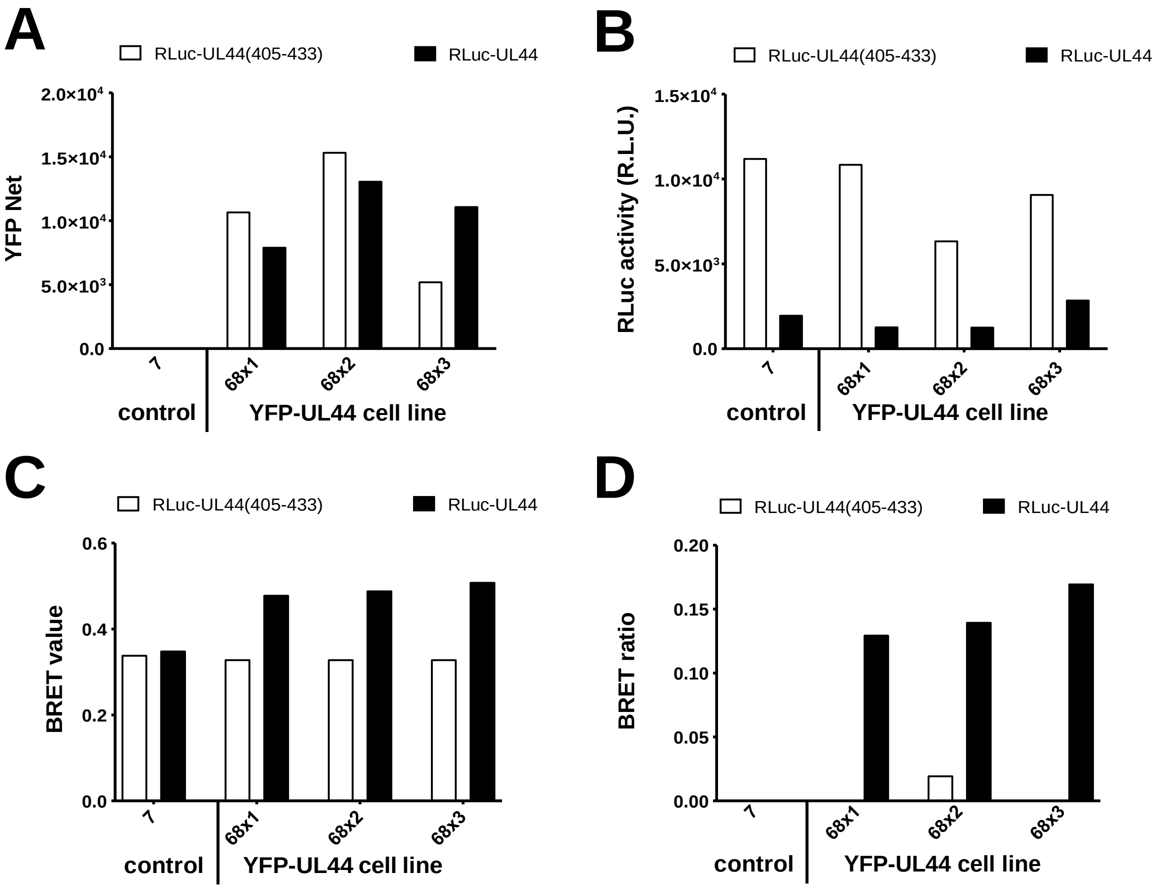

<u>Supplementary Figure S2.</u> **Assessment of YFP-UL44 polyclonal cell lines in BRET assays.** HEKA polyclonal cell lines either transduced with empty vector pWPI-puro (7). or with pWPI-UL44-puro once (68x1), twice (68x2) or trice (68x3) were analyzed in BRET assays after transfection with either RLuc-UL44(405-433; white boxes) or RLuc-UL44 (black boxes). YFP fluorescence (A), RLuc activities (B) as well as the BRET values (C) and BRET ratios (D) relative to each condition are shown.

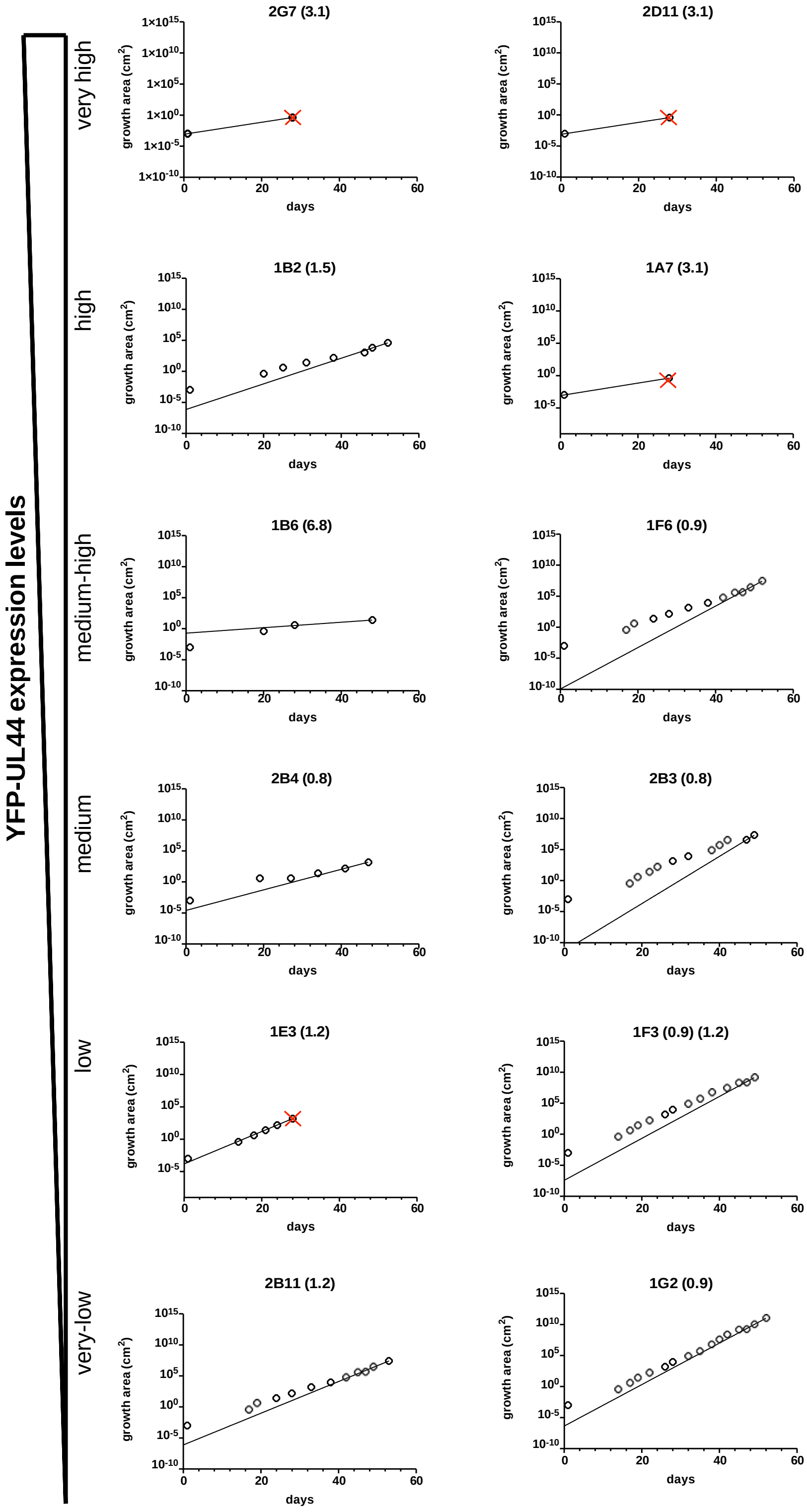

Supplementary Figure S3. Establishment of YFP-UL44 monoclonal cell lines in BRET assays. HEKA polyclonal cell line 68x3 cells were seeded in 96 well plates (0.5 cells/well). Wells containing single cells were monitored daily for cell growth and fluorescence expression under an inverted fluorescence expression. Cells were cultured and passaged when reached confluency. Data shown to the indicated cell lines indicate the approximatively area covered by each cell line at different times post seeding. A red cross indicates cells stopped growing and were therefore discarded. All other cell lines were aliquoted and stored under liquid nitrogen for further usage.
